# LPA2 protein is involved in photosystem II assembly in *Chlamydomonas reinhardtii*

**DOI:** 10.1111/tpj.15405

**Published:** 2021-07-31

**Authors:** Michela Cecchin, Jooyeon Jeong, Woojae Son, Minjae Kim, Seunghye Park, Luca Zuliani, Stefano Cazzaniga, Andrea Pompa, Chan Young Kang, Sangsu Bae, Matteo Ballottari, EonSeon Jin

**Affiliations:** ^1^ Dipartimento di Biotecnologie Università di Verona Strada le Grazie 15 Verona 37134 Italy; ^2^ Department of Life Science Hanyang University 222, Wangsimni‐ro, Seongdong‐gu Seoul 04763 Korea; ^3^ Department of Chemistry Hanyang University 222, Wangsimni‐ro, Seongdong‐gu Seoul 04763 Korea; ^4^ Dipartimento di Scienze Biomolecolari Università degli studi di Urbino Via Aurelio Saffi, 2 Urbino 61029 Italy; ^5^ Istituto di Bioscienze e Biorisorse Consiglio Nazionale delle Ricerche Via Madonna Alta, 130 Perugia 06128 Italy

**Keywords:** photosystem II, *Chlamydomonas reinhardtii*, photosynthesis, chloroplast biogenesis, genome editing, CRISPR

## Abstract

Photosynthetic eukaryotes require the proper assembly of photosystem II (PSII) in order to strip electrons from water and fuel carbon fixation reactions. In *Arabidopsis thaliana*, one of the PSII subunits (CP43/PsbC) was suggested to be assembled into the PSII complex via its interaction with an auxiliary protein called Low PSII Accumulation 2 (LPA2). However, the original articles describing the role of LPA2 in PSII assembly have been retracted. To investigate the function of LPA2 in the model organism for green algae, *Chlamydomonas reinhardtii*, we generated knockout *lpa2* mutants by using the CRISPR‐Cas9 target‐specific genome editing system. Biochemical analyses revealed the thylakoidal localization of LPA2 protein in the wild type (WT), whereas *lpa2* mutants were characterized by a drastic reduction in the levels of D1, D2, CP47 and CP43 proteins. Consequently, reduced PSII supercomplex accumulation, chlorophyll content per cell, PSII quantum yield and photosynthetic oxygen evolution were measured in the *lpa2* mutants, leading to the almost complete impairment of photoautotrophic growth. Pulse‐chase experiments demonstrated that the absence of LPA2 protein caused reduced PSII assembly and reduced PSII turnover. Taken together, our data indicate that, in *C. reinhardtii*, LPA2 is required for PSII assembly and proper function.

## Significance Statement

LPA2 is a nuclear‐encoded protein that is suggested to be involved in PSII assembly in vascular plants. Here, using CRISPSRS/CAS9 mutagenesis, we identified and investigated an LPA2 homolog in *Chlamydomonas reinhardtii*. *lpa2* mutants were characterized by a strong reduction in the accumulation of photosystem II core subunits, reduced photosystem II activity, increased photosensitivity and impairment in photoautotrophic growth, revealing the critical role of LPA2 in the assembly of photosystem II supercomplexes in green algae.

## Introduction

Photosystem II (PSII) is the initial complex in the linear electron transport of photosynthesis in chloroplasts (Nelson and Junge, 2015). It comprises a light‐harvesting antenna complex that absorbs sunlight and a core complex that converts light into photochemical energy (van Amerongen and Croce, 2013; Shen et al., 2019; Su et al., 2017) The PSII core complex contains at least 20 subunits with various cofactors, including electron donors and acceptors (Gokhale and Sayre, 2009). As a result of the structural complexity of PSII, the proper assembly of its subunits is important for its function (Lu, 2016; Nickelsen and Rengstl, 2013).

Although photosynthetic eukaryotes have, through endosymbiosis, acquired chloroplasts that perform oxygenic photosynthesis, the chloroplast genome does not encode all the proteins necessary for the photosynthetic machinery (Shinozaki et al., 1986). Numerous nuclear genes encode components of the photosynthetic apparatus. Moreover, the multiple proteins required for the biogenesis and assembly of protein complexes in the chloroplast, e.g. the CpSRP54, CpSRP43, CpFTSY and LTD proteins from the chloroplast signal recognition particle pathway, are encoded by nuclear genes (Jeong et al., 2017; Jeong et al., 2018; Kirst and Melis, 2014; Ziehe et al., 2017).

The biogenesis of PSII is a stepwise assembly process (Lu, 2016; Nickelsen and Rengstl, 2013). The first step is the formation of the D1 and D2 heterodimer, where the chlorophyll special pair involved in PSII photochemistry is bound (Rokka et al., 2005). Next, the inner antenna proteins CP47 and CP43 are sequentially bound (Boehm et al., 2011). Subsequently, the oxygen‐evolving complex assembles on the lumenal side of the PSII pre‐complex, which is converted into an active monomeric PSII (Bricker et al., 2012; Rokka et al., 2005). Finally, the active PSII forms dimers and is surrounded by the peripheral light‐harvesting antenna complex, which completes the *de novo* biogenesis of PSII (Nickelsen and Rengstl, 2013; Shen et al., 2019; Su et al., 2017).

Many regulatory factors are involved in the appropriate organization of the PSII subunits. Of these, Psb27 in cyanobacteria interacts with CP43 and PSII during both the *de novo* biogenesis and the repair of PSII (Komenda et al., 2012). As two Psb27 homologs have been identified in the green lineage, the role of cyanobacterial Psb27 was proposed to be divided between two genes in eukaryotes (Nickelsen and Rengstl, 2013). One of them, *Psb27‐H2* (*LPA19*), participates in *de novo* PSII assembly by interacting with D1 and CP43 (Wei et al., 2010).

In cyanobacteria, CP43 incorporation into PSII requires another assembly factor, Sll0606, the absence of which results in a drastic reduction in the level of PSII (Zhang et al., 2010). A homolog of Sll0606 is found in the microalga *Chlamydomonas reinhardtii*, but not in the land plant *Arabidopsis thaliana*, suggesting that Sll0606 might be functionally replaced by other proteins in embryophytes (Chi et al., 2012; Nickelsen and Rengstl, 2013). One possible replacement is low PSII accumulation 2 (LPA2), which has been suggested to interact with CP43 during PSII assembly in *A. thaliana*, although this was based on reports that have since been retracted (Cai et al., 2010; Ma et al., 2007). Reduced PSII activity and reduced growth was also reported in *A. thaliana lpa2* mutants in a following work, where LPA2 was shown to interact with the Tellurite resistance C protein (TerC), involved in the insertion of thylakoid membrane proteins (Schneider et al., 2014). LPA2 homologs have been found in other embryophytes, but not in *C*. *reinhardtii* or cyanobacteria (Chi et al., 2012; Nickelsen and Rengstl, 2013). Therefore, CP43 assembly was not expected to require an LPA2 homolog in *C*. *reinhardtii*, but no detailed study of the assembly factors for CP43 in this microalga has been performed. In this study, we identified an LPA2 homolog in the *C*. *reinhardtii* genome. To investigate the function of this protein *in vivo*, we used the ribonucleoprotein (RNP)‐mediated CRISPR‐Cas9 system to generate target‐specific knockout mutants (*lpa2*) of *C*. *reinhardtii*. In the absence of the LPA2 protein, *lpa2* mutants had reduced levels of PSII core subunits and dysfunctional PSII supercomplexes. These results indicate that LPA2 is required for efficient PSII assembly in *C*. *reinhardtii*. In addition, *lpa2* mutants had enhanced electron transport around PSI, suggesting that PSI can be used to dissipate excitation energy in PSII‐deficient conditions.

## Results

### The *LPA2* gene in *Chlamydomonas reinhardtii*

**Figure 1:**
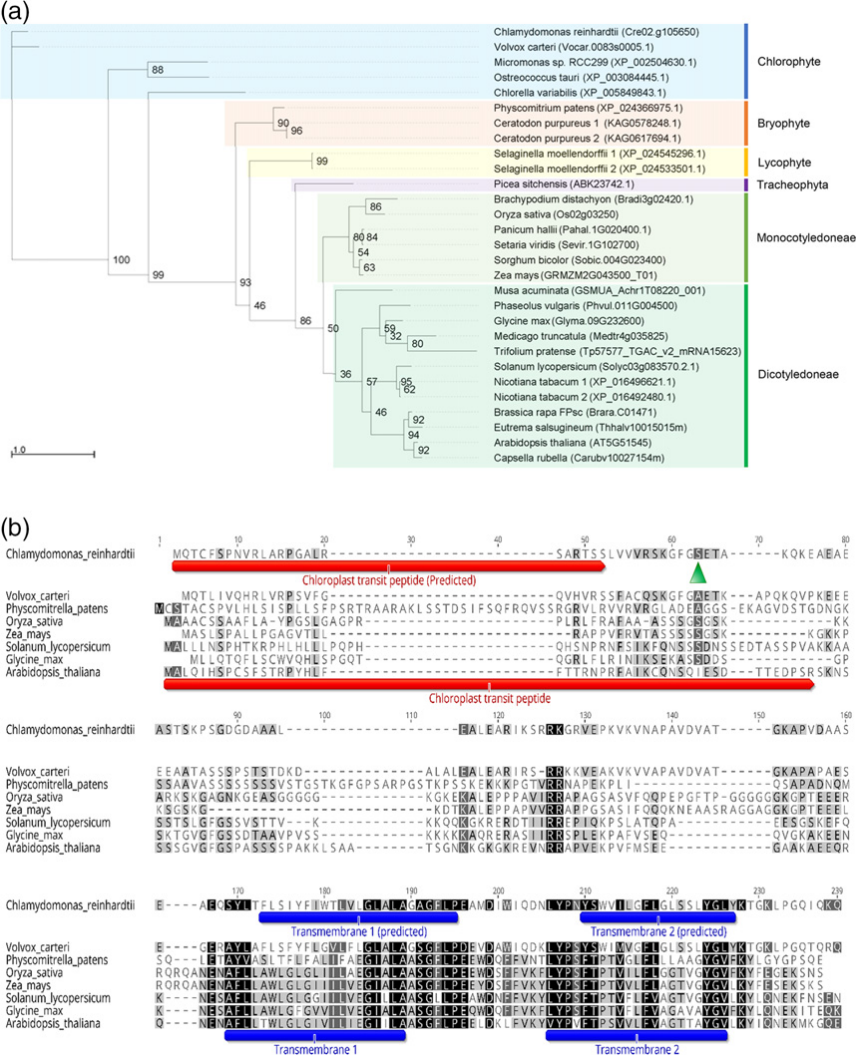
Maximum‐likelihood tree (a) and amino acid sequence alignment (b) of LPA2 homologs in the green lineage. The sequences were aligned using muscle, and selected species representing each clade are shown in (b). Node labels are bootstrap support values from 100 replicates. The species names and accession number of individual sequences are labeled at the tip of the branch. The green triangle represents the Cas9‐driven mutation site of the *LPA2* gene in *lpa2*.

The putative *LPA2* gene (Cre02.g105650) was identified in the *C*. *reinhardtii* genome based on the amino acid sequence similarity between its product and LPA2 in *A. thaliana* (Figure 1). Homologs were also identified in the green lineage, including chlorophytes, but not in cyanobacteria. Moreover, no LPA2 homologs could be found in *Glaucophytes*, *Rhodophyta* or in species derived from secondary endosymbiosis, such as *Cryptophyta*, *Haptophyta* or *Heterokonta*, suggesting that the LPA2 protein is of eukaryotic origin, having evolved in particular in *Viridiplantae* (Figure 1a; Table S1). The *LPA2* gene could be identified also in bryophytes, lycophytes and tracheophytes, but not in hornwort, where no homolog could be found. The absence of the *LPA2* gene in hornwort could be related to some specific evolutionary events that require dedicated and in‐depth analysis. The *C*. *reinhardtii LPA2* gene (*CrLPA2*) encodes a protein of 175 amino acids, including a 24‐amino‐acid‐long chloroplast transit peptide, predicted by predalgo software (http://lobosphaera.ibpc.fr/cgi‐bin/predalgodb2.perl?page=main), and two transmembrane domains (amino acids 109–131 and 146–163), determined by tmhmm software (http://www.cbs.dtu.dk/services/TMHMM/). The CrLPA2 protein shares 23.2% identity and 43.2% similarity with its Arabidopsis homolog (Figure 1b).

To investigate the function of *C*. *reinhardtii* LPA2, we analyzed the light‐inducible expression of *LPA2*. We exposed *C*. *reinhardtii* strain CC503 to a high level of light (500 μmol photons m^−2^ sec^−1^) for 0, 30 and 60 min, and used quantitative reverse transcription polymerase chain reaction (qRT‐PCR) to analyze the transcript levels of *ELIP2* and *LPA2* (Figure S1). Although in the case of *ELIP2* treatment with high light caused a statistically significant increased transcription, *LPA2* expression was only moderately enhanced after exposure to high light. Western blotting revealed the presence of LPA2 in purified chloroplasts, specifically in the thylakoid membranes, but not in the stromal fraction (Figure S1).

### Generation of knockout mutants without the *LPA2* gene in *Chlamydomonas reinhardtii*

**Figure 2:**
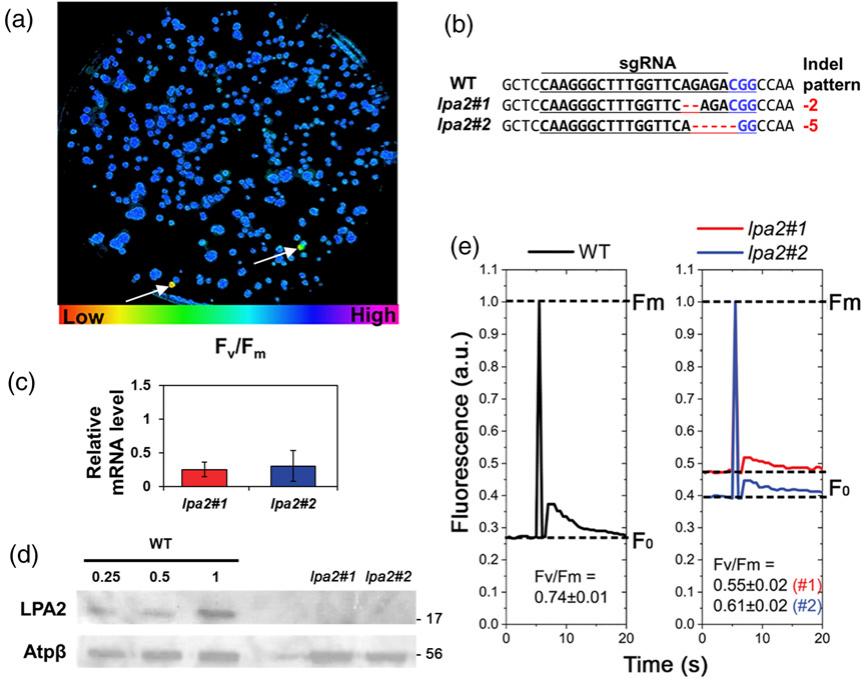
CRISPR‐Cas9‐mediated *lpa2* mutant generation in *Chlamydomonas reinhardtii*. (a) The measurement of *F*_v_/*F*_m_ to select putative *LPA2* gene knockout mutants grown on TAP agar medium under 50 μmol photons m^–2^ sec^–1^. The cells (marked with white arrows) presenting lower *F*_v_/*F*_m_ values than the background cells were picked and confirmed by Sanger sequencing. (b) DNA sequence alignment of the wild type (WT) and *lpa2* mutants obtained from (a) at the *LPA2* locus. The 20‐bp target sequence of sgRNA2 is underlined, and the PAM sequence is shown in blue. The column on the right indicates the number of inserted (+) or deleted (−) bases. (c) Relative mRNA expression of the LPA2 gene in *lpa2* mutants compared with the WT case. mRNA expression levels were calculated based on the WT normalized with the expression level of the *RACK1* gene, used as an internal standard. Error bars are reported as the standard deviation (*n* = 3). (d) Immunoblot with LPA2 and AtpB (loading control) antibodies in the WT and *lpa2#1* and *lpa2#2*, which were used for all further experiments. Proteins were loaded on the basis of equal cell numbers, and the WT samples were loaded at three different concentrations (25, 50 and 100%). (e) The measurement of chlorophyll (Chl) fluorescence kinetics in the WT and *lpa2* mutants grown in liquid TAP medium under 50 μmol photons m^–2^ sec^–1^. The measuring light (ML) and saturating light (SL) were 5 and 1250 μmol photons m^–2^ sec^–1^, respectively. The *F*_v_/*F*_m_ differed significantly between the WT and *lpa2* mutants, as determined by Student’s *t*‐test (*n* = 4; the values shown are means ± SDs; *P* < 0.05).

To further characterize *C*. *reinhardtii LPA2*, we generated target‐specific knockout mutants by using pre‐assembled Cas9 protein small guide RNA (sgRNA) RNP complex‐mediated CRISPR‐Cas9. Different sgRNAs were tested for the generation of *lpa2* mutants, with positive results obtained only in the case of sgRNA2 containing the 5′‐CAAGGGCTTTGGTTCAGAGACGG‐3′ sequence (Table S2). Considering a possible phenotype in the assembly of the pigment binding complexes (Ma et al., 2007), *lpa2* mutant strains were screened on the basis of Chl fluorescence. Transformants with lower *F*_v_/*F*_m_ fluorescence signals than the background cells (Figure 2a) were selected for Sanger sequencing analysis of the target locus. All such transformants had small indels in the *LPA2* gene (Figure 2b). The knockout efficiency, calculated as the ratio of the mutant number (3) to the total colony number (606), was 0.495%, which was similar to the targeted mutation frequency obtained from the total gDNA of CRISPR‐Cas9 transfected cells (0.4%; Table S3). The transcription of the *LPA2* gene was investigated in the *lpa2* mutants, compared with the wild type (WT), revealing reduced transcription in the mutants (Figure 2c). Cas9‐driven mutations occurred at the first exon of the *LPA2* gene, where *lpa2#1* and *lpa2#2* mutants were deleted by 2 and 5 bp, respectively (Figure 2b): we can speculate that these deletions may cause non‐functional transcripts that could be unstable in mutants, as previously reported for other genes edited by Cas9 (Tang et al., 2018; Tuladhar et al., 2019), even if further work is required to support this hypothesis. LPA2 protein accumulation was then investigated by immunoblotting analysis, showing no detectable results in the case of *lpa2* mutant strains (Figure 2d). Analysis of the *lpa2* mutants for potential off‐target effects by targeted deep sequencing revealed no indels (Table S4).

### Decreased chlorophyll (Chl) content in *lpa2* mutants

As revealed during mutant screening, *lpa2* mutants had an aberrant *F*_v_/*F*_m_ fluorescence signal (Figure 2e). Interestingly, although the *F*_m_/Chl ratios of the mutants were similar to that of the wild type, the *F*_0_/Chl ratios of the mutants were increased, resulting in a low *F*_v_/*F*_m_ fluorescence signal. The increased *F*_0_/Chl ratio suggests the partial disconnection of antenna complexes from PSII. The organization of the photosynthetic apparatus was thus investigated on the basis of 77K fluorescence emission spectra. In the case of *lpa2* mutants, the spectra were characterized by an increased fluorescence emission at 680 nm, which can be ascribed to the presence of a disconnected light‐harvesting complex (LHC) protein, confirming the partial destabilization of the PSII complexes (Figure S2). Interestingly, *lpa2* mutants were characterized by increased 715/686 and 715/690 fluorescence emission ratios. Fluorescence emissions at 686 and 690 nm are related to PSII contributions, whereas emission at 715 nm is related to PSI (Girolomoni et al., 2019; Snellenburg et al., 2017): increased 715/686 or 715/690 fluorescence emission ratios in *lpa2* mutants compared with the WT suggests an increased PSI/PSII ratio and/or increased antenna proteins bound to PSI in the mutant strains.

To understand the change in Chl fluorescence caused by the mutation, we analyzed the Chl content of the *lpa2* mutants (Table 1). In photoautotrophic cultures, *lpa2* mutants exhibited approximately 50% reduction in total Chl content per cell compared with that in the WT, whereas the Chl *a*/*b* ratio was not significantly affected. The reduction in Chl content per cell was not related to a change in cell size, which was similar in the different strains analyzed (Table 1).

**Table 1:** Chlorophyll (Chl) content and cell diameter of the wild type (WT) and *lpa2* mutants. *Statistical significance of differences between WT and *lpa2* mutants (*P* < 0.05, *n* = 4), as determined by Student’s *t*‐test

	Chl/cell (pg/cell)	Chl *a*/*b* ratio	Chl/car	Cell diameter (µm)
WT	2.50 ± 0.11	2.61 ± 0.01	3.20 ± 0.03	8.92 ± 0.81
*lpa2#1*	1.35 ± 0.11*	2.58 ± 0.02	2.70 ± 0.07*	8.57 ± 0.96
*lpa2#2*	1.17 ± 0.05*	2.47 ± 0.07	2.82 ± 0.08*	8.39 ± 0.75

### *lpa2* mutants had strongly reduced photoautotrophic growth and reduced photosynthetic activity

**Figure 3:**
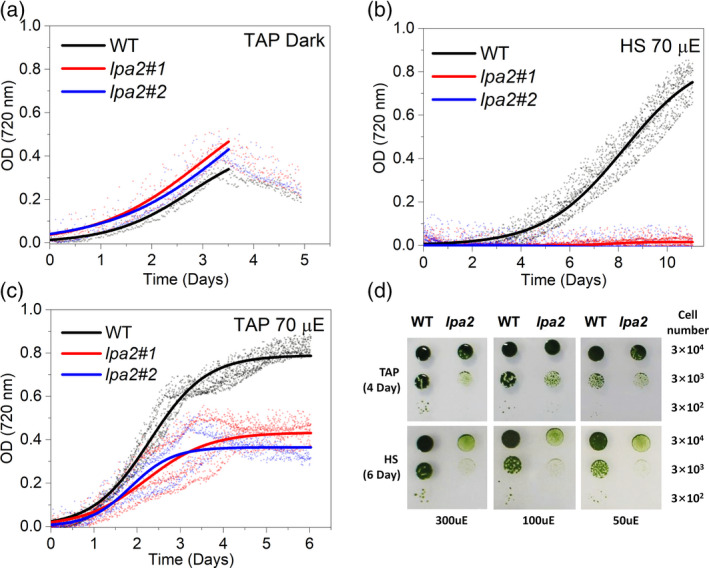
Growth curves of the wild type (WT) and the *lpa2* mutants. Heterotrophic (a), photoautotrophic (b) and mixotrophic (c) growth of the *lpa2* mutants measured in liquid medium and compared with the WT. Heterotrophic conditions were induced by growing microalgae strains in the dark in TAP medium, whereas photoautotrophic and mixotrophic conditions were obtained by growing the cells under continuous light in HS or TAP media, respectively. Growth curves are reported as optical density (OD) measured at 720 nm every 30 min. The growth curves obtained were fitted using the sigmoidal function (*n* = 4). The doubling times of the cells in the different growth conditions are reported in Table 2. Photoautrophic and mixotrophic growth was also evaluated by spot test in solid HS or TAP media at 50, 100 and 300 μmol photons m^–2^ sec^–1^ (d). The number of cells spotted for each drop are reported on the right of (d).

**Figure 4:**
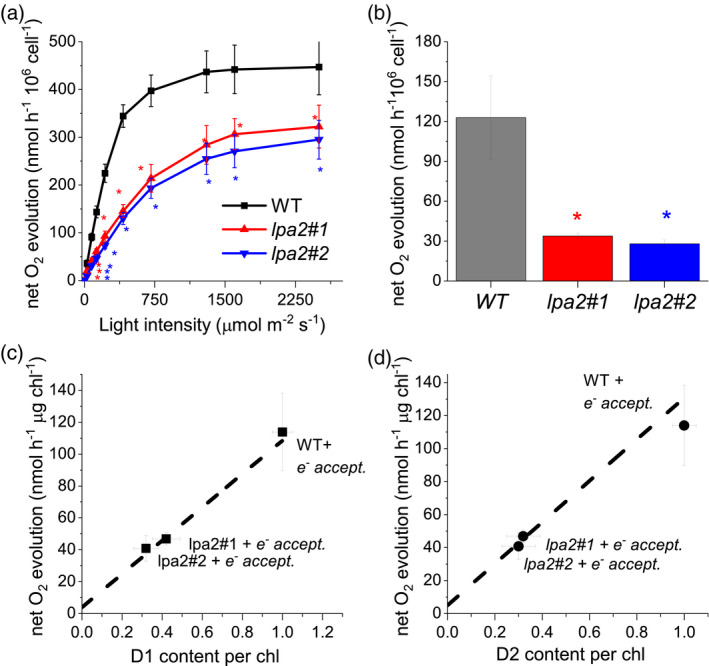
Oxygen evolution curves of the wild type (WT) and the *lpa2* mutants. Oxygen evolution rates of both the WT and the *lpa2* mutants in response to different light intensities were measured to determine the rate of oxygen consumption in the dark. Cells were grown in TAP medium, washed with HS medium and cultivated in photoautotrophy for 12 h prior to measurement. The net oxygen evolution rates were measured on samples at the same cell concentration (a). Oxygen evolution was also measured at 600 μmol photons m^–2^ sec^–1^ in the presence of PSII electron acceptors DCBQ and potassium ferrocyanide (III) and DMBIB as an inhibitor of plastoquinone reduction by cyclic electron flow: the net oxygen evolution rates were normalized to the total cell contents (b). Oxygen evolution rates produced by WT and *lpa2* mutants in the presence of DCBQ, potassium ferrocyanide (III) and DBMIB were then plotted as a function of D1 or D2 content per chlorophyll (Chl) (c, d). Error bars indicate the standard deviation (*n* = 3). The statistical significance of differences between WT and *lpa2* is indicated as *P < 0.05, as determined by Student’s *t*‐test. Results of the linear fitting of the data reported in (c) and (d) are reported as dashed lines (*R* ^2 ^= 0.926 and 0.99545 for the linear fits in c and d, respectively).

To investigate how the reduced *F*_v_/*F*_m_ and reduced Chl content found in the *lpa2* mutants affected their growth, we cultivated WT and mutant strains under photoautotrophic, mixotrophic and heterotrophic conditions. Under heterotrophic conditions (with acetate as a source of organic carbon), the growth of *lpa2* mutants was similar to that of the WT, indicating that the mutations introduced did not affect the light‐independent cell functions. In mixotrophy, *lpa2* mutants showed slower growth than the WT in both solid and liquid media. Under photoautotrophic conditions, the growth of *lpa2* mutants was severely impaired (Figure 3), presumably because the lower photosynthetic activity of the mutants could not maintain whole‐cell metabolism under these conditions. Interestingly, in the case of *lpa2* mutants the doubling time in the exponential phase was similar in mixotrophy and heterotrophic conditions, suggesting that the growth rate in TRIS acetate phosphate (TAP) medium was essentially driven by acetate consumption in this mutant strain (Table 2). Consistent with this finding, *lpa2* mutants were essentially not replicating in the time range analyzed in photoautotrophic growth conditions. In contrast, WT cells grown in mixotrophy were characterized by a reduced doubling time compared with both heterotrophic and photoautotrophic conditions. The photosynthetic activity of *lpa2* mutants was then analyzed using pulse‐amplitude modulated (PAM) fluorescence (Figure S3). The operating efficiency of PSII electron transport (ΦPSII) was lower in the mutants than in the WT at light intensities below 400 μmol m^–2^sec^–1^ but was similar at higher irradiances (Figure S3). The fraction of excitation energy not used for the photochemical reaction could be lost through safe non‐photochemical reactions, leading to controlled energy conversion into heat (ΦNPQ) or uncontrolled dissipation (ΦNO), which is usually related to oxidative stress and photoinhibition. The controlled thermal dissipation of the absorbed excitation energy (ΦNPQ) was also lower in the *lpa2* mutants than in the WT, whereas the fraction of absorbed energy lost by uncontrolled dissipation (ΦNO) was higher in *lpa2* (Figure S3). Accordingly, the NPQ values, calculated as (*F*_m_ –*F*_m_′)/*F*_m_′, were lower in the *lpa2* mutants than in the WT, implying a lower photoprotective capacity in the mutants (Figure S3). The fraction of closed PSII centers, calculated from the 1 – *q*_L_ value (Kramer et al., 2004), was similar in the *lpa2* mutants compared with that in the WT (Figure S3). This result indicates that, despite the reduced efficiency of PSII, the redox state of the primary quinone acceptor (Q_A_) was maintained similar to that in the WT under different light intensities owing to the acclimation of the overall photosynthetic apparatus. We further investigated PSII activity by measuring the light‐dependent oxygen evolution curves and found reduced oxygen evolution on a per‐cell basis in the *lpa2* mutants, confirming its reduced photosynthetic activity (Figure 4a). In order to investigate PSII activity specifically, oxygen evolution was measured in the presence of an electron acceptor for plastoquinones 2,6‐dichloro‐1,4‐benzoquinone (DCBQ), and its secondary acceptor potassium ferricyanide (III), in the presence of inhibitor DBMIB, preventing any possible influence of PSI on the plastoquinone redox state (Böhme, 1976; Brinkert et al., 2016). As presented in Figure 4(c), reduced PSII activity was measured in the presence of DCBQ, potassium ferricyanide (III) and DBMIB in *lpa2* mutants. Interestingly, in presence of an electron acceptor for plastoquinones, the light‐dependent net oxygen evolution was linearly correlated with the level of D1 or D2 (Figure 4d). This result suggests that lower oxygen evolution was linked with decreased PSII accumulation in the mutants.

**Table 2:** Doubling times of the wild type (WT) and *lpa2* mutant strains. Doubling times (h) for the WT and the *lpa2* mutants were calculated for cells in the exponential phase with mixotrophic, photoautotrophic and heterotrophic growth conditions, as reported in Figure 3. *Statistical significance of differences between WT and *lpa2* mutants (*P* < 0.05, *n* = 3), as determined by Student’s *t*‐test

	Mixotrophy	Autotrophy	Heterotrophy
	TAP + LIGHT	HS + LIGHT	TAP + DARK
WT	7.8 ± 0.4	19.4 ± 0.3	18.3 ± 1.0
*lpa2#1*	14.6 ± 0.7*	388.3 ± 8.7*	15.4 ± 1.5
*lpa2#2*	11.8 ± 1.1*	406.5 ± 9.1*	18.3 ± 1.5

### The *lpa2* mutants have enhanced electron transport flow around PSI

**Figure 5:**
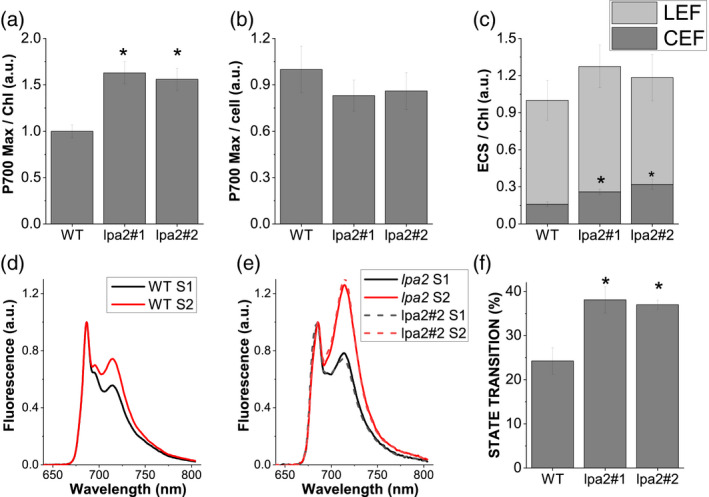
PSI activity, electrochromic shift (ECS) and state transitions. (a, b) Maximal P700 oxidation on a chlorophyll (Chl) basis (a) or cell basis (b) in the wild type (WT) and the *lpa2* mutants. (c). Linear electron flow (LEF) and cyclic electron flow (CEF) of the WT, *lpa2*#1 and *lpa2#2* estimated from the ECS on a Chl basis. (d, e) State transitions analysis using 77 000 fluorescence emission spectra of the WT (d) and *lpa2*#1 and *lpa2#2* mutants (e) in state 1 (S1) or state 2 (S2) conditions. (f) Maximum level of state transition measured as percentage variation of PSII fluorescence in S2 compared with S1. Error bars are indicated as the standard deviation (*n* = 3). *Statistical significance of differences between WT and *lpa2* mutants (*P* < 0.01), as determined by Student’s *t*‐test.

The activity of PSI was measured as maximum P700 oxidation, which was higher on a Chl basis in the *lpa2* mutants than in the WT (Figure 5a), but was similar on a per cell basis because of the reduced Chl content per cell in the mutants (Figure 5b). These results suggest that the defect in PSII activity increased PSI activity on a Chl basis in the *lpa2* mutants.

Next, we measured the electrochromic shift (ECS) to estimate the proton‐motive force (*pmf*) across the thylakoid membranes generated by the light‐driven electron flux. The *pmf* in the *lpa2* was similar to that in the WT (Figure 5c). Considering the reduced PSII activity in the *lpa2* mutants, we investigated the possible influence on cyclic electron flow (CEF) around PSI on *pmf* by measuring the ECS in the presence of DCMU to inhibit linear electron flow. The *lpa2* mutants had an increased fraction of *pmf* related to CEF (Figure 5c), causing a similar total *pmf* despite the decreased PSII activity.

We reasoned that the altered levels of PSII activity in the *lpa2* mutants might affect the state transitions that balance the energy between PSI and PSII by using LHCII migration from PSII to PSI. We examined the capacity of the *lpa2* mutants to perform state transitions by measuring the 77K fluorescence emission spectra of cells under the conditions of state 1 or state 2 (Figure 5d,e and f). The *lpa2* mutants showed an increased migration of light‐harvesting antenna proteins to PSI under state‐2 conditions, suggesting an increased pool of mobile LHCII subunits, likely because of the reduced PSII assembly.

### The *lpa2* mutants accumulate low levels of PSII core subunits

**Figure 6:**
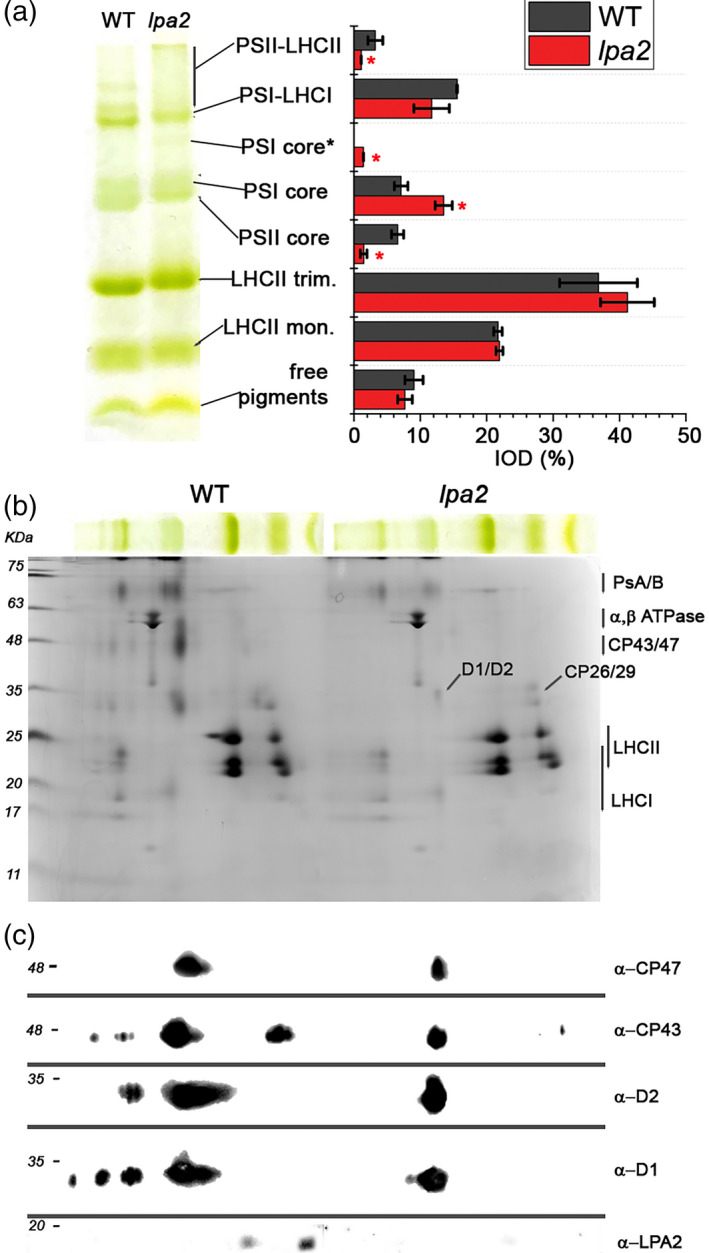
The 2D Deriphat SDS‐PAGE of purified thylakoid membranes. (a) Deriphat PAGE of the wild type (WT) and the *lpa2* mutants. The density of each band was quantified by densitometric analysis of the green channel of the picture. Band marked with PSI core* is related to PSI core with residual Lhca antenna bound. The second dimension of the 2D Deriphat SDS‐PAGE was performed by running the Deriphat PAGE lanes in Tris‐Tricine poly‐acrylamide gel. *Statistical significance of differences between WT and *lpa2* (*P* < 0.05), as determined by Student’s *t*‐test (*n* = 3). (b) Western blot analysis on 2D Deriphat SDS‐PAGE by using specific antibodies recognizing D1, D2, CP43 or CP47 subunits, reported in (c).

We investigated the effect of the *LPA2* gene mutation on the organization of photosynthetic complexes in isolated thylakoid membranes (Figure 6). In the 2D Deriphat SDS‐PAGE analysis, the intensity of the bands representing the PSII core and the PSII supercomplexes was markedly reduced in the *lpa2* mutant, with a particularly strong decrease in the CP43/CP47 band and, although to a lower extent, in the D1/D2 band. Interestingly, the LPA2 protein was detected in the WT as a monomer and at higher oligomerization state, likely interacting with other proteins involved in PSII assembly, as previously reported in the case of *A. thaliana* (Ma et al., 2007; Schneider et al., 2014).

**Figure 7:**
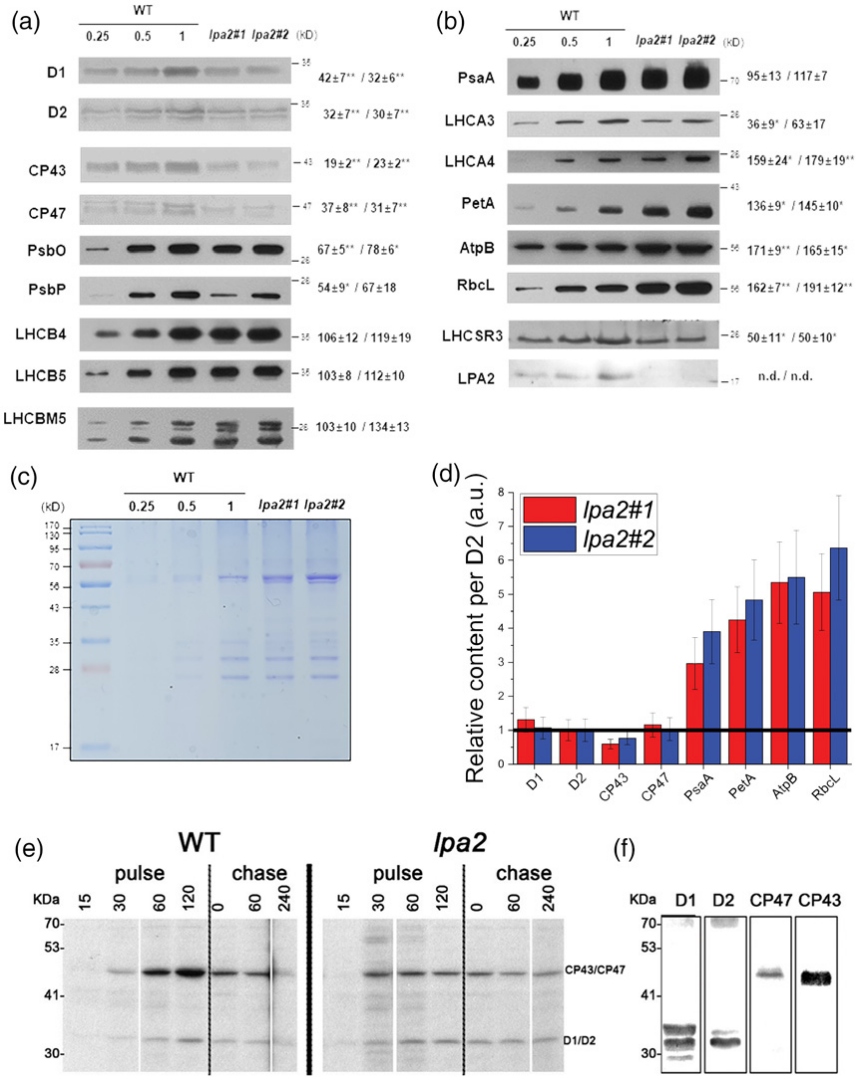
Immunoblot analysis (a, b) and Coomassie blue‐stained SDS‐PAGE gel (c) of chloroplast proteins from the wild type (WT) and the *lpa2* mutants. Results from immunoblotting with antibodies against PSII–LHCII are shown in (a), where specific antibodies recognizing the PSII core subunits D1, D2, CP43, CP47, PsbO and PsbP and the LHCII antenna complexes LHCB4 (CP29), LHCB5 (CP26) and LHCBM were used. For LHCBM complexes in particular, the antibody LHCBM5 was adopted, which was reported to recognize all of the different LHCBM subunits. Results from immunoblotting with antibodies against PSI core (α‐PsaA), LHCI antenna (α‐LHCA3, α‐LHCA4), Cyt *f* (α‐PetA), chloroplast ATPase β‐subunit (α‐AtpB), large subunit of RUBISCO (α‐RbcL) and LHCSR3 are shown in (b). Each lane was loaded on a per chlorophyll (Chl) basis (1 μg), and the WT samples were loaded at three different concentrations (0.25, 0.5 and 1 μg). The levels of proteins in the *lpa2#1* and *lpa2#2* mutants compared with the WT are presented next to the protein bands expressed as percentages of the WT level (*n* ≥ 3; the values shown are means ± SDs). The statistical significance of differences between WT and *lpa2* is indicated as *P < 0.05 and **P < 0.01, determined by Student’s *t*‐test. In (d), the protein content of D1, D2, CP43 CP47, PsaA, PetA, AtpB and RUBISCO was normalized to the D2 content, with the different ratios set as 1 in the case of the WT (black line). (e) Autoradiography of immunoprecipitated PSII complexes. *Chlamydomonas reinhardtii* WT and *lpa2* mutant cells were grown in TAP medium under low light (70 µmol m^−2^sec^−1^) in the presence of [^35^S] methionine and [^35^S] cysteine with the different times (pulses) indicated above the figure in minutes. PSII core complexes were extracted from thylakoid membranes by membrane solubilization and immunoprecipitation using the D2 antibody. Chase experiments were performed after 60 min of pulse removing the labelled amino acids from the growth medium. The bands corresponding to D1, D2, CP43 and CP47 were identified according to their migration pattern, as reported in (f), where immunoblotting results were obtained on thylakoid membranes isolated from the WT and loaded on the same SDS‐PAGE gel system as in (e).

Western blot analyses of specific photosystem subunits (Figure 7) reveal, on a Chl basis, the strongest decrease in CP43, with approximately 20% residual CP43 in the *lpa2* mutants, followed by CP47, D1 and D2, which were reduced to approximately 30–40%, compared with the WT. A significantly reduced accumulation of other PSII core subunits, such as PsbO and PsbP, was also detected in the case of *lpa2* mutants (Figure 7). The accumulation of LHCII complexes in the *lpa2* mutants was similar to that in the WT on a Chl basis, indicating that the LHCII/PSII ratio in the mutants was increased. Considering the low NPQ measured in the case of the *lpa2* mutants, the accumulation of LHCSR3, the main Chl binding protein involved in this photoprotective mechanism, was also investigated (Peers et al., 2009). As reported in Figure 7, an approximately 50% reduction of LHCSR3 was measured on a Chl basis in the absence of LPA2. LHCSR proteins have been reported to be involved in quenching mechanisms occurring at the level of PSII, disconnected LHCII and PSI‐LHCI (Cazzaniga et al., 2020; Dinc et al., 2016; Girolomoni et al., 2019): the ratio LHCSR3/PSII was similar in the WT and *lpa2* mutants, whereas a reduced LHCSR3/LHCII and LHCSR3/PSI was evident in the *lpa2* mutants.

PSI accumulation on a Chl basis was not affected by the *LPA2* mutation (Figure 7), with a consequent increase in the PSI/PSII ratio in the *lpa2* mutants compared with that in the WT. Interestingly, in the case of LHCI a possible reorganization of different Lhca subunits is likely to have occurred in *lpa2* mutants, as evinced by the different content of subunits recognized by α‐Lhca3 and α‐Lhca4 antibodies in the *lpa2* mutants, which were decreased and increased, respectively, on a Chl basis compared with the WT. It has already been reported that the quality of the Lhca complexes bound by PSI can be modulated according to different growth conditions (Bonente et al., 2012): the destabilization of PSII observed in *lpa2* is thus likely to induce acclimation mechanisms at the level of PSI.

The accumulation of the cytochrome *b*_6_ *f* complex and ATP synthase was investigated using Western blotting with antibodies specific to cytochrome *f* and the ATPase β‐subunit, respectively (Figure 7). The levels of both subunits were significantly increased on a Chl basis in the *lpa2* mutant. The large Rubisco subunit, a representative enzyme of the Calvin–Benson cycle, was clearly increased in the mutants on a Chl basis. As reported in Figure 7(d), the reduced PSII content on a Chl basis was accompanied in *lpa2* mutants by a strong increase of the cytochrome *b*_6_ *f* complex, PSI, Rubisco and ATP synthase content per PSII. On a cell basis, with the reduced Chl content observed in the case of *lpa2* mutants, the strong decrease in PSII subunits in these strains was accompanied by a reduction of PSI and cytochrome *b*_6_ *f* complex, and a similar content of Rubisco and ATP synthase, compared with the WT (Figure S4).

In order to evaluate whether the absence of LPA2 protein specifically affected PSII assembly or its turnover rate, we performed pulse‐chase experiments, followed by thylakoid solubilization and PSII core immunoprecipitation with D2 antibodies. As reported in Figure 7(e), upon SDS‐PAGE separation of proteins co‐immunoprecipitated by D2 antibody, two main bands appeared, at approximately 45 and 35 kDa, respectively: the former band can be attributed to CP43 and/or CP47, whereas the latter band can be attributed to D2, probably with the co‐migration of the subunit D1 (Figure 7f). The *lpa2* mutants were characterized by a similar incorporation of D1/D2 in the PSII complex, compared with the WT, in the time range analyzed here. In contrast, a strongly reduced CP43/CP47 assembly was evident after a 60‐ or 120‐minute pulse, suggesting a key role for LPA2 in PSII assembly. After 1 h of chase in low light, the D1/D2 and CP47 contents were strongly reduced in the WT because of its high turnover rate and assembly of new label‐free complexes, but not in the *lpa2* mutants, indicating a slower PSII turnover, likely as a consequence of the partially impaired assembly.

### PSII photosensitivity and D1 repair in *lpa2* mutants

**Figure 8:**
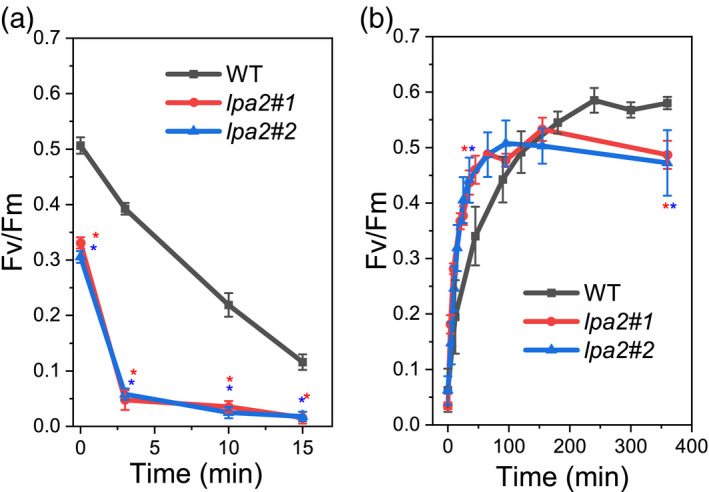
Kinetics of PSII activity photoinhibition and repair. The kinetics of PSII photoinhibition (a) and repair (b) were measured following the changes in maximum quantum yield (*F*_v_/*F*_m_) after exposure to strong light (1800 µmol m^–2^ sec^–1^). (a) Kinetics of PSII activity (*F*_v_/*F*_m_) photoinhibition. (b) Kinetics of *F*_v_/*F*_m_ recovery under low light (15 µmol m^–2^ sec^–1^) after exposure to strong light (1800 µmol m^–2^ sec^–1^) for 15 min in the case of *lpa2* mutants and 25 min in the case of the wild type (WT). Error bars are indicated as the standard deviation (*n* = 3). *Statistical significance of differences between WT and *lpa2* mutants (*P* < 0.05), as determined by Student’s *t*‐test.

PSII complexes that are not fully assembled are highly unstable and more sensitive to high light treatment, which causes photooxidation (Fu et al., 2007). We monitored the level of D1 protein during exposure to high light (500 µmol m^–2^ sec^–1^) in the presence or absence of lincomycin, a chloroplast protein biosynthesis inhibitor (Figure S5). In the *lpa2* mutant, the relative level of D1 protein decreased faster than in the WT when they were shifted from low light to high light, suggesting an increased photosensitivity of PSII complexes in the mutants. To explore the photosensitivity of PSII in *lpa2* mutants and the potential role of the LPA2 protein in repairing the D1 subunit, we performed light‐shift experiments and monitored the rate of photoinhibition and recovery. After exposure to strong light (1800 µmol m^–2^ sec^–1^), the *F*_v_/*F*_m_ values were remarkably reduced in *lpa2* mutants (Figure 8a), with a considerably faster rate than that of the WT, suggesting a strong photosensitivity in the absence of the LPA2 protein. Upon a shift to low light (15 µmol m^–2^ sec^–1^), PSII repair mechanisms were activated: the PSII repair occurred faster in the *lpa2* mutants (Figure 8b). Over a longer time period, PSII repair in the WT was more effective, leading to the restoration of higher *F*_v_/*F*_m_ values than those in *lpa2* mutants. These results are consistent with the reduced CP43/CP47 incorporation in PSII complexes resulting from pulse experiments on a longer time scale (with 60‐ or 120‐minute pulses).

## Discussion

Here, we demonstrate that in contrast to a previous report (Ma et al., 2007), an LPA2 homolog is present in the model organism for green algae *C*. *reinhardtii*. Moreover, we found LPA2 homologs in different *Chlorophytes* but not in cyanobacteria or in other eukaryotic algae species, suggesting that LPA2 evolved specifically in eukaryotes belonging to the green lineage. Therefore, to expand our understanding of PSII biogenesis and assembly in green algae, we used the CRISPR‐Cas9 methodology to investigate this nuclear‐encoded protein, LPA2, and elucidate its role in PSII biogenesis via photochemical and biochemical analysis of two independent *lpa2* mutants.

### The lack of LPA2 affects PSII assembly in *Chlamydomonas reinhardtii*

The lack of LPA2 protein, which is localized in the thylakoid membranes, resulted in a strong reduction of the growth of *lpa2* mutants in mixotrophy conditions and an almost complete impairment of growth in photoautotrophy (Figure 3). Similar results were obtained previously in *C*. *reinhardtii* mutants depleted of CP43 (Marín‐Navarro et al., 2007; Zerges et al., 2003): in the case of *lpa2* mutants the CP43 subunit was observed to be reduced to approximately 20% of the WT level. In the absence of LPA2 protein, other PSII core subunits were also decreased, with an approximately 60–70% reduced accumulation of CP47, D1 and D2 on a Chl basis in *lpa2* mutants (Figure 7). Pulse‐chase experiments showed that, in the absence of LPA2, the assembly of D1/D2 complexes into PSII occurs at a similar rate compared with the WT. By contrast, in the case of CP43/CP47, assembly into PSII was essentially saturated in *lpa2* mutants in the first 30 min but was further increased in the WT, even after a 120‐min pulse (Figure 7e). The thylakoidal LPA2 protein is thus involved in the assembly of CP43/CP47 into PSII, which also contributes to the stabilization of the overall PSII core complex. It is worth noting that PSII assembly proceeds through the formation of a D1/D2/CP47 complex, followed by the incorporation of CP43: LPA2 could be specifically involved in the assembly of CP43 into PSII, as previously suggested for *A. thaliana* in the retracted work of Ma et al. (2007). Alternatively, LPA2 could be involved in both CP43 and CP47 assembly into PSII: additional research efforts are required to discriminate between these two different hypotheses.

PSII in t3Dhe *lpa2* mutants showed increased photosensitivity (Figures 8 and S5) at high light intensity. D1 is the PSII component that is the most sensitive to excess light energy (Melis, 1999; Nickelsen and Rengstl, 2013); photodamaged D1 is rapidly replaced with newly synthesized D1 (Järvi et al., 2015; Lu, 2016; Nickelsen and Rengstl, 2013). For D1 replacement, PSII is partially disassembled by the detachment and reassembly of CP43 (Järvi et al., 2015; Lu, 2016; Nickelsen and Rengstl, 2013). Previous work has demonstrated that in *C*. *reinhardtii de novo* PSII assembly and D1 repair are distinct mechanisms occurring in different regions of the chloroplast (Uniacke and Zerges, 2007). Indeed, despite the slower PSII turnover rate observed in the case of *lpa2* mutants during chase experiments, D1 repair was enhanced over the shorter time scale in the mutants upon strong light exposure (Figure 8). The faster recovery of PSII activity in *lpa2* mutants (Figure 8b) could be related to an increased activation of the D1 repair mechanism as an acclimation response through the increased photosensitivity of the partially assembled PSII complexes occurring in the absence of LPA2 protein. In *A. thaliana*, LPA3 is a major factor required for CP43 detachment during D1 repair; no evidence suggests that LPA2 is involved in PSII repair (Chi et al., 2012; Järvi et al., 2015). Our D1 regeneration results in *C*. *reinhardtii lpa2* mutants indicate that indeed the involvement of LPA2 in PSII repair is limited and related to longer time scales, whereas the main role of this protein is in the *de novo* biogenesis of PSII.

### The lack of LPA2 affects the accumulation of the photosynthetic machinery

The absence of the LPA2 protein increased the *F*_0_/Chl ratio and caused an increased contribution of the 77 000 fluorescence emission spectra at 680 nm in the *lpa2* mutant, indicating that the efficiency of the excitation energy transfer from the antenna complex to the reaction center is reduced because of the partial disconnection of the PSII–LHCII supercomplex (Figures 2e and S2). Likewise, native Deriphat PAGE showed a lower level of the PSII–LHCII supercomplex in the mutant, if any, but the unconnected LHCII remained as a free antenna (Figure 6). A large decrease in CP43 and CP47 in the mutants implies that *C*. *reinhardtii* LPA2 participates in their accumulation, as these subunits are an important link between the PSII reaction center and the antenna complex. The reduced PSII assembly in *lpa2* mutants caused a reduction of PSII activity (Figure 4), which contributed to the reduced growth phenotype observed in the *lpa2* mutants (Figure 3), which became even more severe under photoautotrophic conditions, causing the whole‐cell metabolism to rely upon photosynthesis. The increase in the ΦNO of the mutants also indicated insufficient photoprotective regulation of energy dissipation (Figure S3), in agreement with the increased photosensitivity of PSII in these mutants and posing a serious problem for *lpa2* mutant survival without a carbon source (Figure 3). One of the main photoprotective mechanisms in green algae, NPQ, was reduced in *lpa2* mutants despite the trigger for this mechanism, and proton accumulation in the lumen (Peers et al., 2009) was similar compared with the WT, as demonstrated by the ECS measurements (Figure 5c). Accordingly, LHCSR3 content was reduced in the absence of LPA2 protein (Figure 7), but the ratio between LHCSR3 and PSII was similar in WT and *lpa2* mutants. However, it is worth noting that LHCSR proteins have been reported to function as a quencher also at the level of disconnected LHCII (Dinc et al., 2016) and PSI‐LHCI antenna (Girolomoni et al., 2019; Kosuge et al., 2018), with LHCSR3/LHCII and LHCS3/PSI ratios being reduced in the *lpa2* mutants (Figure 7). Moreover, LHCSR3 expression and maximum NPQ activity should be investigated in high light, whereas *lpa2* mutants presented a reduced growth phenotype even at low light (Peers et al., 2009). Further work is required to investigate more deeply the reason for the low NPQ phenotype of *lpa2* mutants. Another mechanism previously reported to be involved in photoprotection in *C*. *reinhardtii* is the shift in balance of excitation pressure between PSI and PSII through state transitions (Allorent et al., 2013). The increase state transition capacity of *lpa2* mutants could be a consequence of the reduced PSII activity, inducing a migration of antenna proteins toward PSI. Alternatively, the reduced formation of the PSII–LHCII supercomplex might lead to an increased fraction of mobile LHCII, resulting in an increased capacity for state transitions.

Unlike PSII, the abundance of PSI core subunits and PSI activity were not reduced by the lack of LPA2 protein (Figures 5 and 7), with a consequent increase of the PSI/PSII ratio in the mutant. These findings differ from those of previous studies showing lower PSI activity in mutants defective in PSII biogenesis (Wang et al., 2013; Zhang et al., 2011), including the case of *lpa2* mutants in *A. thaliana* (Ma et al., 2007). These features suggest that in *C*. *reinhardtii* the *lpa2* mutants might preferentially operate PSI‐mediated electron transport flow to release excitation pressure and generate trans‐thylakoid proton transport to compensate for the inactive PSII. Indeed, the fraction of CEF, which is critical in maintaining the *pmf*, was higher in the *lpa2* mutants than in the WT (Figure 5).

The imbalance between PSII and PSI in the *lpa2* mutants resulted in another interesting phenotype: the over‐accumulation on a Chl basis of the cytochrome *b*_6_ *f* complex and Rubisco, which are involved in downstream photosynthetic reactions (Figure 7d). Similarly, ATP synthase content was increased in *lpa2* mutants, suggesting a possible faster relaxation of lumen acidification. However, the observed reorganization of the photosynthetic apparatus in the *lpa2* mutants is not sufficient to sustain photoautotrophic growth, probably because of the reduced PSII‐dependent linear electron flow, causing a consequent reduced NADPH formation and increased photosensitivity.

### Comparison between LPA2 functions in *Chlamydomonas reinhardtii* and *Arabidopsis thaliana*

Comparing the effect of *lpa2* mutation in *C*. *reinhardtii* with the previous retracted results reported for *A. thaliana* (Ma et al., 2007), several features were shared, such as severely reduced growth, reduced PSII assembly, strong reduction in PSII core subunit accumulation, reduced PSII activity and photochemical efficiency, and an increase in Cyt *f* content and ATPase (Ma et al., 2007). However, in the case of *C*. *reinhardtii* an increased PSI/PSII ratio and increased P700 activity on a Chl basis were evident in the *lpa2* mutant, whereas in *A. thaliana* both PSI accumulation and P700 activity were lower compared with the WT. In the absence of LPA2 protein causing reduced PSII assembly, the cell acclimation mechanisms were thus somewhat different in *C*. *reinhardtii* compared with *A. thaliana*, with a specific increase in PSI activity in *C*. *reinhardtii*. Moreover, here we report increased RUBISCO content, increased state transitions, increased CEF and increased D1 repair, but reduced NPQ capacity, in *lpa2* mutants of *C*. *reinhardtii*, whereas to our knowledge similar acclimation events in *lpa2* mutants of *A. thaliana* have not yet been investigated.

In conclusion, the knockout mutation of *LPA2* in *C*. *reinhardtii* resulted in a drastic reduction in the level of PSII, with a concomitant decrease in its efficiency. In the absence of the thylakoidal LPA2 protein, not only were accumulations of CP43, CP47, D1 and D2 strongly reduced, but the residual PSII was more prone to photoinhibition, leading to increased D1 repair on a short time scale. However, further studies are required to understand the detailed mechanism through which LPA2 plays this role. The process of PSII protein assembly is complicated, and the functions of various assembly factors are almost certainly coordinated. Some of these factors, such as LPA1/rep27, PAM68 and Alb3, which function in the same step, could form a protein complex (Armbruster et al., 2010); thus, building a protein interaction network that can provide a comprehensive view of the interplay among different assembly factors, repair complexes and PSII subunits is necessary. PSII assembly factors such as Alb3 and PAM68 emerged early in the evolution of photosynthetic organisms because they are present in all cyanobacterial groups, green algae and embryophytes (Chi et al., 2012). Conversely, LPA2 is present in the green lineage (Figure 1), but no homolog of LPA2 has been identified in the cyanobacterium *Synechocystis* PCC6803, red algae, glaucophytes and algae that contain a red algal plastid, suggesting that LPA2 appeared in the green plant lineage (Chi et al., 2012).

It is worth noting that during the revision process of this work, a preprint was released reporting a role of LPA2 in PSII assembly in *C*. *reinhardtii*, essentially confirming the results described herein (Spaniol et al., 2021).

## Experimental Procedures

### Sequence alignments and phylogenetic analysis

LPA2 homologs were identified in the National Center for Biotechnology Information (NCBI) non‐redundant protein sequences database using BlastP. The sequences obtained showed a Blast query coverage of more than 50% of the alignment and at least 40% amino acid identity with LPA2 of *A. thaliana* or *C*. *reinhardtii*. Additionally, we searched more homolog sequences in the DOE‐JGI Phytozome proteome database using Blast and selected sequences that matched the hidden Markov models profile with an *e* value of <10^−3^ (Potter et al., 2018). The species and accession numbers of LPA2 homologs were summarized in Table S1. The LPA2 homolog was not found in Glaucocystophyceae, Rhodophyta, Cryptophyceae, Haptista, Rhizaria, Stramenopiles and Alveolata. The amino acid sequences were aligned using muscle with the default settings of geneious r10 (Edgar, 2004) and the non‐conserved regions of the alignment were trimmed manually using mesquite 3.61 (http://www.mesquiteproject.org). A maximum‐likelihood tree was constructed using phyml3 with 100 bootstrap analysis (Guindon et al., 2010). The Whelan and Goldman substitution model was selected assuming an estimated proportion of invariant sites and four gamma‐distributed rate categories by smart model selection (Lefort et al., 2017; Whelan and Goldman, 2001). The obtained tree was visualized and edited using archaeopteryx (http://www.phylosoft.org/archaeopteryx).

### CRISPR‐Cas9‐driven mutagenesis

All procedures were performed according to Baek et al. (2016) by using 100 μg of Cas9 protein and 70 μg of gRNA. After CRISPR‐Cas9 transformation, cells were incubated in TAP liquid medium supplemented with 40 mm sucrose for 12 h and harvested for genotype characterization or immediately diluted (to 2 × 10^3^ cells) and plated on TAP medium containing 1.5% agar to obtain single colonies. The colonies were screened on the basis of the *F*_v_/*F*_m_ fluorescence signal by using a Walz Imaging PAM System (M‐series; Heinz Walz GmbH, https://www.walz.com). To confirm the mutation of the target site, we further analyzed the putative mutants by using Sanger sequencing.

### Genotype characterization

Genomic DNA was extracted as described by Jeong et al. (2018). For Sanger sequencing, the target regions were PCR‐amplified by using specific primers (5′‐GTAGTGTGCTTACATTTGCTGATT‐3′ and 5′‐CTACTGCTTCTGGATCTGTCC‐3′ for the *lpa2* gene locus). The PCR products were separated by agarose gel electrophoresis, eluted from the gel, and sequenced (Macrogen, https://www.macrogen.com). For targeted deep sequencing, genomic DNA segments that encompassed the nuclease target sites were amplified using Phusion polymerase (New England Biolabs, https://international.neb.com). Equal quantities of PCR amplicons were subjected to paired‐end read sequencing by using the Illumina MiSeq platform. The next‐generation sequencing data obtained were analyzed using Cas‐Analyzer (Park et al., 2017). Reads that occurred only once were excluded to remove errors associated with amplification and sequencing. Insertions and deletions (indels) located around the Cas9 cleavage site (3 bp upstream of the protospacer‐adjacent motif sequence) were considered to be induced mutations by Cas9. The targeted mutation efficiency was calculated from the mutation counts and the total counts of the reads. To examine the occurrence of potential off‐target mutation sites, we used Cas‐OFFinder (Bae et al., 2014), which lists potential off‐target sites with a DNA or RNA bulge in length that differ from the on‐target sites by up to four nucleotides.

### Pigment and cell size analysis

Pigment analyses were performed on cells grown in TAP medium at 70 μmol photons m^–2^ sec^–1^ by HPLC, as described by Lagarde et al. (2000). Cell size was investigated using the Countless^®^ II FL automated cell counter (ThermoFisher Scientific, https://www.thermofisher.com).

### Growth conditions

The *C*. *reinhardtii* strains were grown in minimal (HS) medium or in the presence of acetate (TAP medium; Kropat et al. 2011). Photoautotrophic and mixotrophic growth were evaluated in 80‐mL photobioreactors in a multi‐cultivator system (Photon System Instruments, https://psi.cz) by growing different strains in either HS or TAP medium in continuous light at 70 μmol photons m^–2^ sec^–1^. Heterotrophic growth was evaluated in cells grown in TAP medium in the dark. Growth curves were retrieved from optical density (OD) measurements at 720 nm automatically acquired from the multi‐cultivator system every 30 min. Doubling times were calculated from the exponential phase of the growth curve, as described by Harris (2009). The spot test was performed by spotting cells grown in TAP medium at 70 μmol m^−2^sec^−1^ in the exponential phase. In particular, 3 × 10^2^, 3 × 10^3^ and 3 × 10^4^ cells were spotted in TAP or HS medium with 1% agar added; plates were then exposed to 50, 100 or 300 μmol m^−2^sec^−1^ for 4 or 6 days, respectively, for cells in TAP or HS media.

### 2D Deriphat SDS‐PAGE electrophoresis and Western blots

The 2D Deriphat SDS‐PAGE analysis was performed as described by Jeong et al. (2018). Thylakoid membranes isolated according to (Ferrante et al., 2012) from cells in exponential phase grown in TAP medium in continuous light at 70 μmol photons m^–2^ sec^–1^. Isolated thylakoids were solubilized at a Chl concentration of 0.5 mg mL^–1^ with *n*‐dodecyl‐α‐d‐maltoside (final concentration, 0.75% for both wild type and *lpa2*), incubated on ice for 10 min and centrifuged at 20 000 ***g*** for 10 min to remove unsolubilized material. Thylakoid membrane proteins (25 µg Chl) were loaded in each lane. After separation, one‐dimensional native Deriphat PAGE strips were cut and soaked in SDS‐PAGE stacking buffer containing 5 m urea twice for 25 min each. The proteins were then separated using SDS‐PAGE (12% gel containing 2 m urea). The acrylamide gels were stained with Coomassie blue. Immunoblot analysis for profiling chloroplast proteins was performed with cells in the exponential phase grown in TAP medium under continuous light at 70 μmol photons m^–2^ sec^–1^, as described by Jeong et al. (2018). Antibodies were purchased from Agrisera (α‐D1, AS05084; α‐D2, AS06146, α‐CP43, AS111787; α‐CP47, AS04038; α‐PsbO, AS06142‐33; α‐PsbP, AS06142‐23; α‐LHCB4, AS06117; α‐LHCB5, AS09407; α‐LHCBM5, AS09408; α‐LHCSR3, AS142766; α‐PsaA, AS09408; α‐PetA, AS01005; α‐AtpB, AS05085‐10; α‐RbcL, AS03037; Agrisera, https://www.agrisera.com), except for Lhca3 and Lhca4 antibodies, which were provided by Prof. Hippler (Jeong et al., 2018; Petroutsos et al., 2011) and the LPA2 antibody. Polyclonal antibodies for LPA2 protein were raised against two peptides: CGFGSETAKQKEAEAEASTSKP and EALEARIKSRRKGRVEPKVKVC (AdipoGen® Life Sciences, https://adipogen.com). In the case of α‐LHCBM5, it is important to note that the antibody recognizes not only LHCBM5 but all the different LHCBM subunits in *C*. *reinhardtii*, as previously described (Girolomoni et al., 2017).

### Photosynthetic activity analysis

Photosynthetic activity of WT and mutant strains was measured in cells in the exponential growth phase grown in TAP medium under continuous light at 70 μmol photons m^–2^ sec^–1^. Before the measurements, cells were washed with HS medium and cultivated in photoautotrophy for 12 h. The PSII activity was analyzed by conducting fluorescence measurements on whole cells using a Dual‐PAM 100 instrument (WALZ, https://www.walz.com). In particular, ΦPSII, ΦNO, ΦNPQ and NPQ were measured in dark‐adapted samples (1 h) using different actinic lights, ranging from 0 to 1700 μmol photons m^–2^ sec^–1^. The 77 000 fluorescence emission spectra were acquired using a charge‐coupled device spectrophotometer (JBeamBio), as previously described (Allorent et al., 2013). State transitions were measured on whole cells induced to state 1 or 2, as described by Fleischmann et al. (1999): in brief, state 1 (S1) was induced by shaking cells vigorously under low light (5 μmol m^2^ sec^−1^) with 10 µm of DCMU for at least 15 min to oxidize the plastoquinone pool; state 2 (S2) was induced by adding 250 μm sodium azide to inhibit mitochondrial respiration and to reduce the plastoquinone pool. The P700 activity was measured using the Dual‐PAM 101 following the kinetics of transient absorption at 830 nm after exposure to actinic light. The maximum P700 activity was measured after a pulse of saturating light. Electrochromic shift measurements were performed using a Photosynq that set the actinic light at 500 μmol photons m^–2^ sec^–1^. Light‐dependent O_2_ evolution curves were measured using a Clark electrode, as reported by Perozeni et al. (2019). Light‐dependent O_2_ evolution was also measured at 600 μmol photons m^–2^ sec^–1^ in the presence of an artificial PSII electron acceptor 2,6‐Dichloro‐1,4‐benzoquinone (DCBQ) at 0.25 mm, 1 mm potassium ferricyanide (III) and 1 mm dibromothymoquinone (DBMIB) (Brinkert et al., 2016). PSII repair kinetics were measured after exposure to strong light (1800 μmol photons m^–2^ sec^–1^) until the *F*_v_/*F*_m_ values were reduced to 0.05. PSII regeneration was then induced in low light (15 μmol photons m^–2^ sec^–1^) by measuring *F*_v_/*F*_m_. The *F*_v_/*F*_m_ values were measured after 3 min of dark adaptation of whole cells.

### Pulse‐chase and immunoprecipitation

Immunoprecipitation experiments were performed in cells at the exponential phase grown in TAP medium under continuous light at 70 μmol photons m^–2^ sec^–1^, as described by De Marchis et al. (2018) for *Nicotiana tabacum* (tobacco) protoplasts, with minor modifications. In brief, approximately 3 million algae cells were subjected to pulse labelling for up to 2 h by using Pro‐Mix – a mixture of [^35^S]Met and [^35^S]Cys (GE Healthcare, https://www.gehealthcare.com). After the pulse, the chase was performed by adding unlabeled Met and Cys to final concentrations of 10 and 5 mm, respectively. Cells were sampled at different pulse and chase time points. The cells were homogenized by adding homogenization buffer (150 mm Tris‐Cl, pH 7.5, 150 mm NaCl, 1.5 mm EDTA, 2% Triton X‐100 and complete protease inhibitor cocktail; Roche, https://www.roche.com) to frozen samples. Proteins were immunoselected using rabbit polyclonal antisera against D2. The immunoprecipitates were analyzed using SDS‐PAGE. After electrophoresis, gels were treated with Amplify™ fluorography reagent (GE Healthcare), dried and exposed for fluorography.

### The *de novo* biosynthesis of D1 protein

To block the translation of the chloroplast‐encoded D1 protein, we added lincomycin, an inhibitor of plastid protein biosynthesis, to the cultures, as described by Jin et al. (2003), and the cells were incubated in TAP medium under either normal growth light (50 μmol photons m^–2^ sec^–1^) or high light (500 μmol photons m^–2^ sec^–1^). Cells were harvested at 0, 30, 60 and 90 min after the light treatment, and the cell pellets were resuspended in Laemmli sample buffer (Laemmli, 1970) without bromophenol blue. After vigorous vortexing, the protein content of the crude extracts was measured using Bradford reagent (Bio‐Rad, https://www.bio‐rad.com).

### RNA expression analysis

Total RNA was isolated from high light‐treated cells in TAP medium by using an RNeasy Plant Mini Kit (Qiagen, https://www.qiagen.com). Total RNA (1 µg) was used as a template for cDNA synthesis by using SuperScript III reverse transcriptase (ThermoFisher Scientific). Next, the cDNA was used as a template to amplify *PsbC* with real‐time PCR by using SYBR Premix Ex Taq II (TaKaRa, https://www.takarabio.com) and a Thermal Cycler Dice Real Time System (TaKaRa). The relative quantities of the transcript were normalized to those of the constitutively expressed *RACK1* gene. The following primer sequences were used for the amplification: 5′‐CAAGAACGTCGTGCTGCTGAA‐3′ and 5′‐CCTGCGTGCCATAAGTGACC‐3′ for ELIP2 (Cre09.g393173); 5′‐CAACTACAGCTGGGTGATCCT‐3′ and 5′‐AGTGTCCAGCTCCCTTTCAG‐3′ for LPA2; and 5′‐GGCTGGGACAAGATGGTCAA‐3′ and 5′‐GAGAAGCACAGGCAGTGGAT‐3′ for RACK1 (Cre06.g278222).

## Author Contributions

EJ and MB designed and moderated the research. SB coordinated the generation of the mutant strains. MC, JJ, MK, AP, WS, LZ, SC, SP, CB and SB performed the experiments. MC, JJ, MB and EJ drafted the article. All the authors analyzed and contributed to the data interpretation.

## Conflict of Interest

The authors declare that they have no conflicts of interest associated with this work.

## Supporting information


**Figure S1**. Gene expression and localization of LPA2 in *C*. *reinhardtii*.
**Figure S2**. 77 000 fluorescence emission spectra of wild type (WT) and *lpa2* mutant.
**Figure S3**. Light intensity response curves of fluorescent photosynthetic parameters.
**Figure S4**. Accumulation of photosynthetic proteins per cell in *lpa2* mutants.
**Figure S5**. Time course analysis for the loss of the D1 protein after a shift from low light to high light.

**Table S1**. Accession numbers of LPA2 homologs used in the phylogenetic analysis.
**Table S2**. Target sequences of sgRNA used to recognize the *lpa2* gene.
**Table S3**. Mutation (insertion and deletion; indel) frequency of wild‐type and RGEN‐transfected cells for each sgRNA.
**Table S4**. Analysis of off‐target effects in the wild type and *lpa2* mutant.

## Data Availability

All the data described herein are included in the Figures or in the supporting information. The strains investigated here are fully available upon request to corresponding authors.
