# Supplementary material for: LPA2 protein is involved in photosystem II assembly in Chlamydomonas reinhardtii
Source: Plant J. 2021 Jul 31;107(6):1648–62. doi: 10.1111/tpj.15405 (PMC8518032; doi:10.1111/tpj.15405)
Supplement: Supplementary file 1 — Figure S1. Gene expression and localization of LPA2 in C. reinhardtii. Figure S2. 77 000 fluorescence emission spectra of wild type (WT) and lpa2 mutant. Figure S3. Light intensity response curves of fluorescent photosynthetic parameters. Figure S4. Accumulation of photosynthetic proteins per cell in lpa2 mutants. Figure S5. Time course analysis for the loss of the D1 protein after a shift from low light to high light. [file TPJ-107-1648-s001.pdf]

SUPPLEMENTARY FIGURES

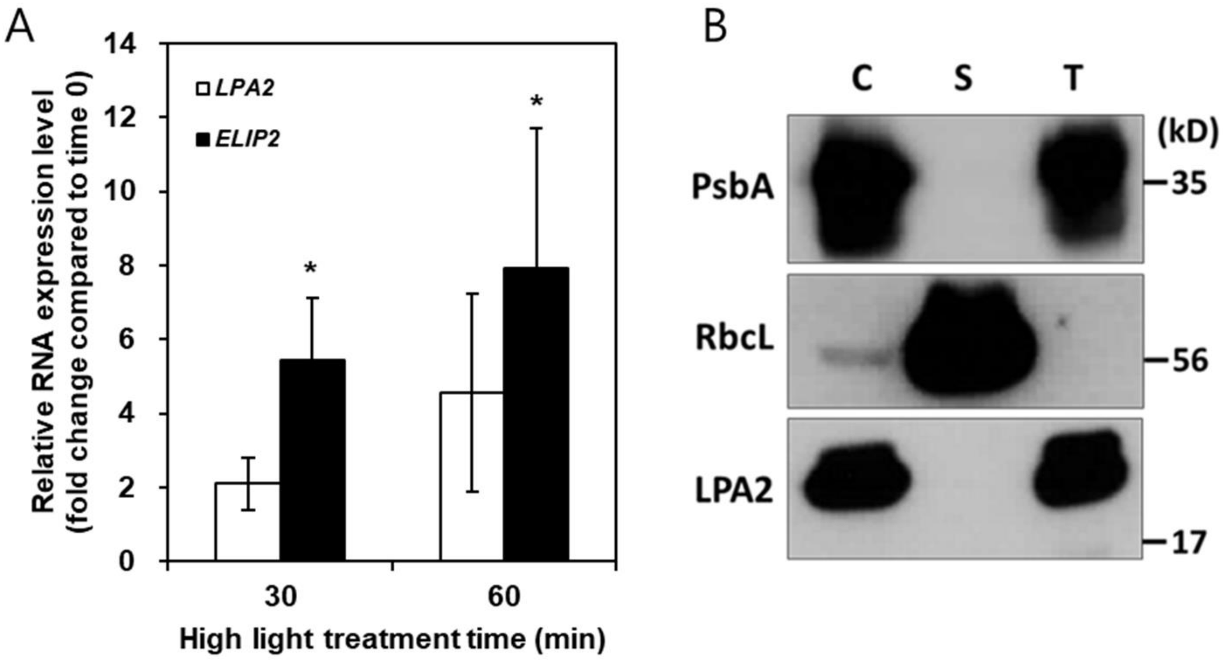

Figure S1: Gene expression and localization of LPA2 in *C. reinhardtii*. A. RNA expression analysis of *LPA2* gene with *ELIP2* (Cre09.g393173) in *Chlamydomonas reinhardtii*. qRT PCR was performed with the RNAs extracted from wild type cells exposed to high light (500 μmol photons m^{-2} s^{-1}) for 0, 30, and 60 minutes. The relative RNA expression level was calculated based on the level obtained at 0 min (value =1) and the values shown are the fold change compared to time 0 upon normalization to the internal control RACK 1. Error bars are reported as standard deviation (n=3). The statistical significance of differences between 0 time to 30 min and 60min are indicated as *, as determined by the Student t-test (*$p < 0.05$). B. Immunoblot analysis of wild type *C. reinhardtii* fractions. C, chloroplast fraction; S, stroma fraction; T, thylakoids fraction. Proteins were loaded on an equal protein basis (30 μg).

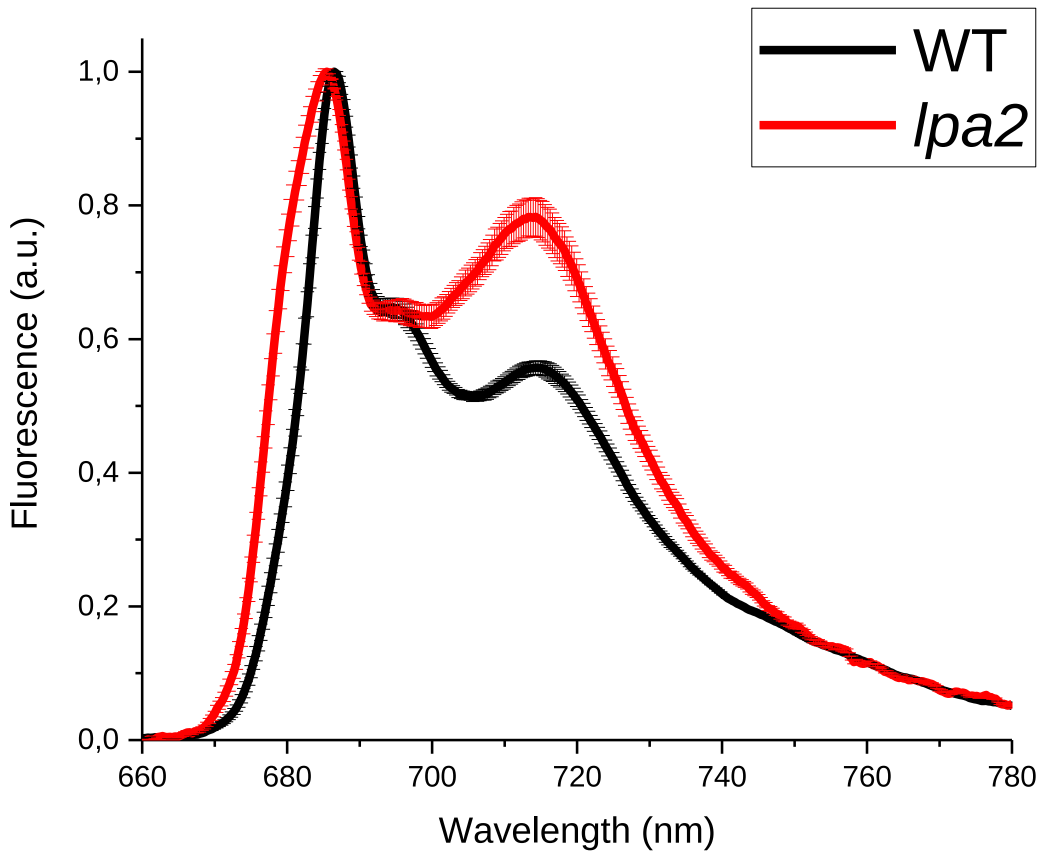

Figure S2. 77K fluorescence emission spectra of wild type (WT) and *lpa2* mutant. Fluorescence emission spectra are reported in the case of lpa2#1 mutant. Similar results were obtained in the case of lpa2#2 mutant, as reported in Figure S4. Cells grown in TAP medium were washed with HS medium and grown in photoautotrophy for 12 hours. Before the measurements cells were incubated in the dark on a shaker for one hour. Fluorescence emission spectra were normalized to the maximum peak (at 686.5 nm in the case of WT and 685.5 nm in the case of lpa2 mutant). Error bars are reported as standard deviation (n=3). The statistical significance of differences at 680 nm and 715 nm between WT and *lpa2* were verified by Student *t*-test obtaining p-values respectively of 0.001 and 0.003.

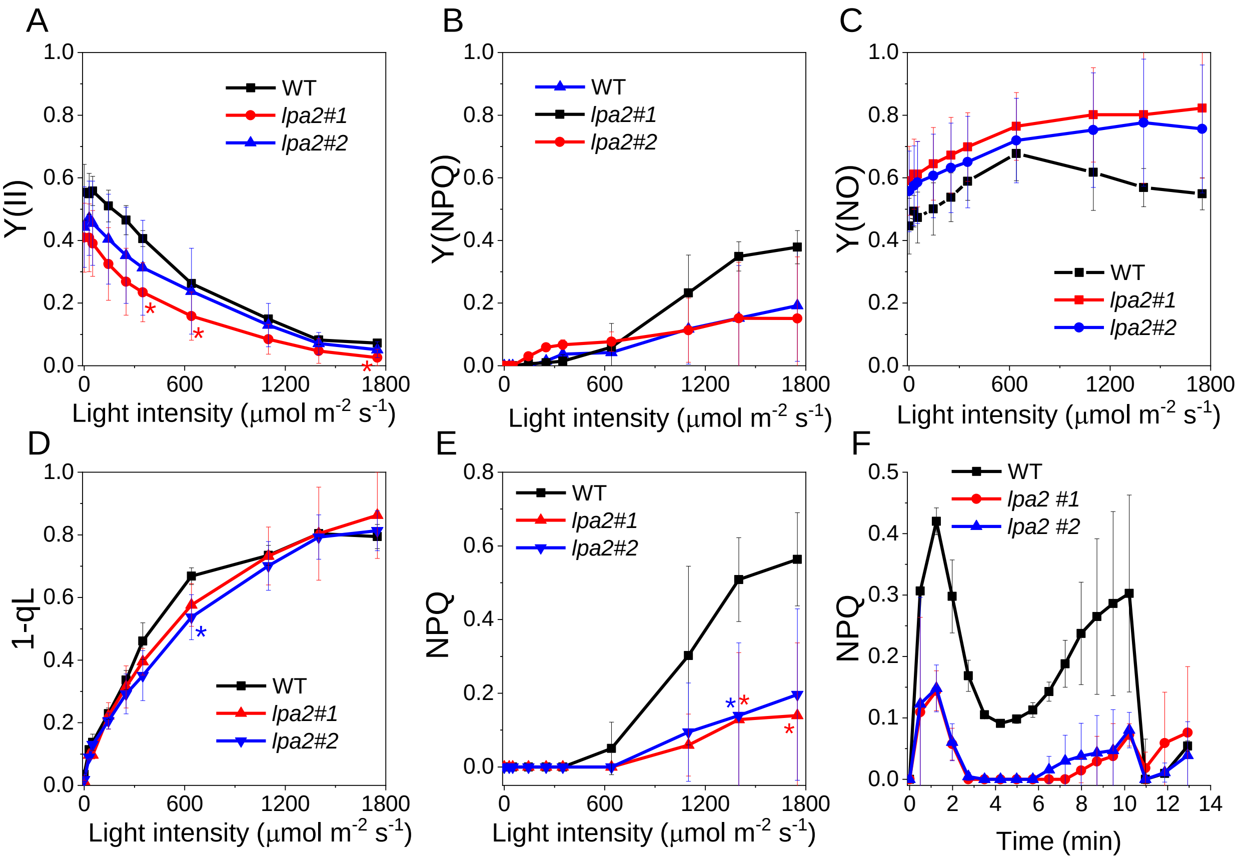

Figure S3. Light intensity response curves of fluorescent photosynthetic parameters. Intensity response curves for φPSII, φNPQ, and φNO in the wild-type (WT), *lpa2#1*, and *lpa2#2* mutants are shown in **A**, **B**, and **C**, respectively. NPQ and 1-q_L are reported in **D** and **E**. Kinetics of NPQ induction and recovery are reported in Panel F: NPQ induction were measured with an actinic light of at 1200 μmol m^{-2} s^{-1} for 10 minutes followed by dark recovery for 3 minutes. Error bars indicate standard deviation (n = 3). The statistical significance of differences between WT and *lpa2* mutants ($p < 0.05$) is indicated as *, as determined by Student *t*-test.

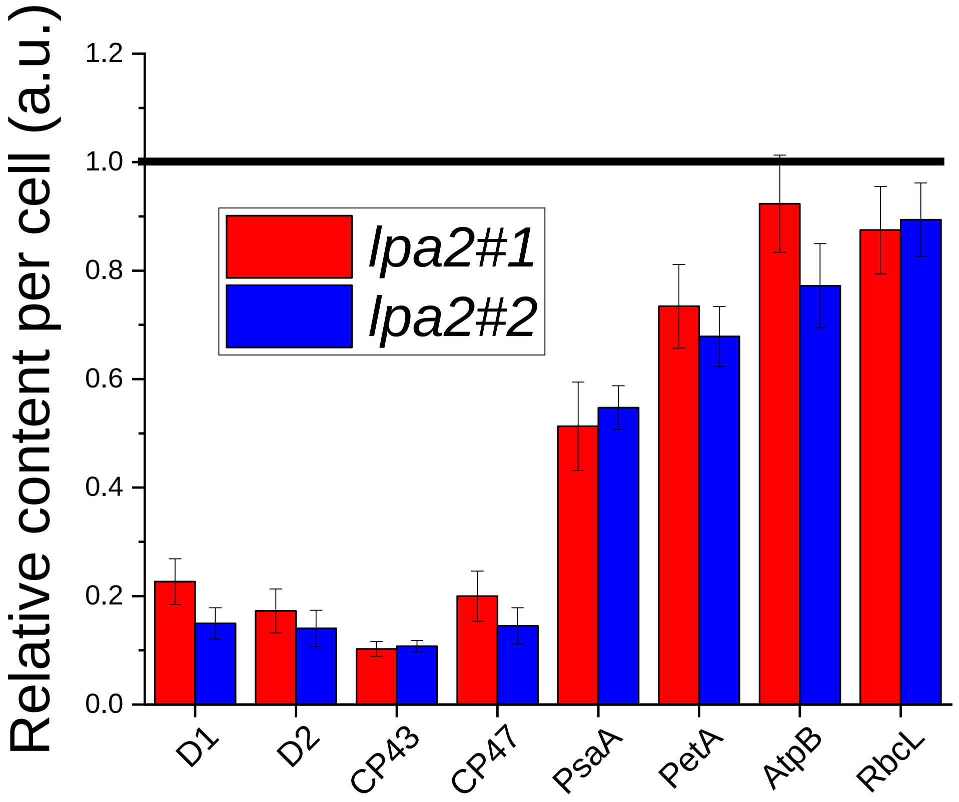

Figure S4. Accumulation of photosynthetic proteins per cell in *lpa2* mutants. Immunoblotting results reported in Figure 7 were obtained loading the same chlorophyll content on SDS-PAGE gel. Here, on the base of the Chl/cell ratio of WT and lpa2 mutants, the protein content on a cell of D1, D2, CP43 CP47, PsaA, PetA, AtpB and RUBISCO are reported in the case of lpa2 mutants. Black line indicate the protein content on a cell basis in the case of WT, set to 1 for the different proteins herein investigated.

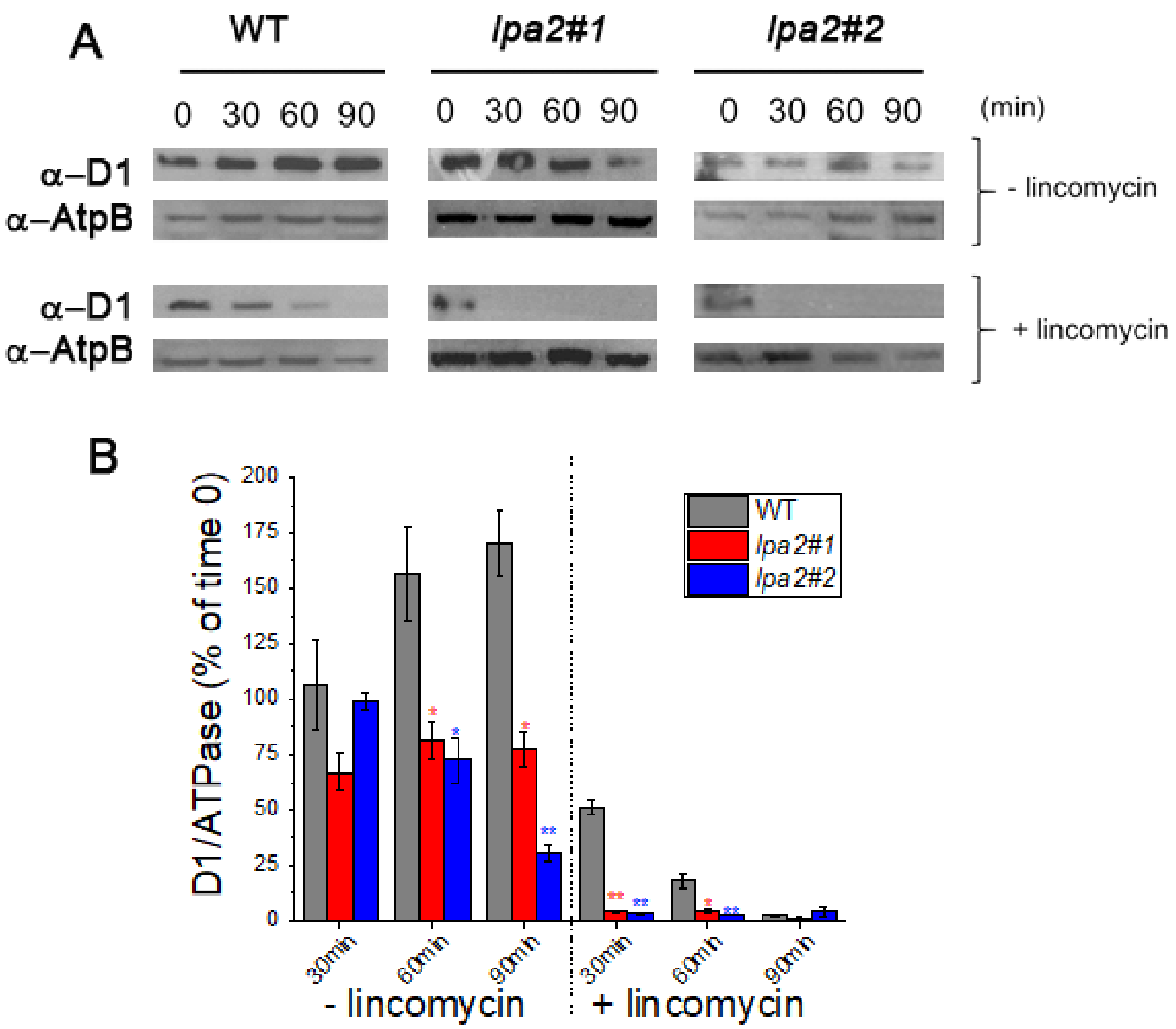

Figure S5. Time course analysis for the loss of the D1 protein after a low light to high light shift. Cells grown in TAP medium in low light (70 µmol photons m^{-2} s^{-1}) were transferred to high light (500 µmol photons m^{-2} s^{-1}) in presence (+) or absence (-) of 5 mM lincomycin. Immunoblots (A) were probed with anti-D1 proteins and anti-AtpB proteins. The results obtained were quantified by densitometry and the data obtained: D1/AtpB ratios were calculated for each time point as expressed as percentage of D1/ATP measured at time 0 in presence or absence of lincomycin (B). Error bars indicate standard deviation (n = 2). The statistical significance of differences between WT and *lpa2* mutants at the different time points is indicated as ** for $p < 0.01$ or * for $p < 0.05$, as determined by Student *t*-test.
