# Supplementary material for: LPA2 protein is involved in photosystem II assembly in Chlamydomonas reinhardtii
Source: Plant J. 2021 Jul 31;107(6):1648–62. doi: 10.1111/tpj.15405 (PMC8518032; doi:10.1111/tpj.15405)
Supplement: Supplementary file 2 — Table S1. Accession numbers of LPA2 homologs used in the phylogenetic analysis. Table S2. Target sequences of sgRNA used to recognize the lpa2 gene. Table S3. Mutation (insertion and deletion; indel) frequency of wild‐type and RGEN‐transfected cells for each sgRNA. Table S4. Analysis of off‐target effects in the wild type and lpa2 mutant. [file TPJ-107-1648-s002.pdf]

SUPPLEMENTARY TABLES

Table S1. Accession numbers of LPA2 homologs used in phylogenetic analysis.

Species	Group	Gene bank/Phytozome accession number
Chlamydomonas reinhardtii	Chlorophyte	Cre02.g105650
Volvox carteri	Chlorophyte	Vocar.0083s0005.1
Micromonas sp. RCC299	Chlorophyte	XP_002504630.1
Ostreococcus tauri	Chlorophyte	XP_003084445.1
Chlorella variabilis	Chlorophyte	XP_005849843.1
Physcomitrium patens	Bryophyte	XP_024366975.1
Ceratodon purpureus 1	Bryophyte	KAG0578248.1
Ceratodon purpureus 2	Bryophyte	KAG0617694.1
Selaginella moellendorffii 1	Lycophyte	XP_024545296.1
Selaginella moellendorffii 2	Lycophyte	XP_024533501.1
Picea sitchensis	Tracheophyta	ABK23742.1
Brachypodium distachyon	Monocotyledoneae	Bradi3g02420.1
Musa acuminata	Monocotyledoneae	GSMUA_Achr1T08220_001
Oryza sativa	Monocotyledoneae	Os02g03250
Panicum hallii	Monocotyledoneae	Pahal.1G020400.1
Setaria viridis	Monocotyledoneae	Sevir.1G102700
Sorghum bicolor	Monocotyledoneae	Sobic.004G023400
Zea mays	Monocotyledoneae	GRMZM2G043500_T01
Arabidopsis thaliana	Dicotyledoneae	AT5G51545
Brassica rapa FPsc	Dicotyledoneae	Brara.C01471
Capsella rubella	Dicotyledoneae	Carubv10027154m
Eutrema salsugineum	Dicotyledoneae	Thhalv10015015m
Glycine max	Dicotyledoneae	Glyma.09G232600
Medicago truncatula	Dicotyledoneae	Medtr4g035825
Nicotiana tabacum 1	Dicotyledoneae	XP_016496621.1
Nicotiana tabacum 2	Dicotyledoneae	XP_016492480.1
Phaseolus vulgaris	Dicotyledoneae	Phvul.011G004500
Solanum lycopersicum	Dicotyledoneae	Solyc03g083570.2.1
Trifolium pratense	Dicotyledoneae	Tp57577_TGAC_v2_mRNA15623

Table S2. Target sequences of sgRNA used to recognize the *lpa2* gene.

	RGEN target (5' to 3')	Position	Direction	GC content (%, w/o PAM)	Out-of-frame score	Mismatches				
						0	1	2	3	4
sgRNA1	GTTGTCCGCTCCAAGGGCTTTGG	79	+	60	59.4	1	0	0	0	8
sgRNA2	CAAGGGCTTTGGTTCAGAGACGG	90	+	50	74.4	1	0	0	0	5
sgRNA3	GCAAGCACCTCCAAGCCGTCGGG	139	+	65	50.2	1	0	0	2	17
sgRNA4	CAAGGGGCGTGTTGAGCCCAAGG	216	+	65	62.7	1	0	0	0	11

Table S3. Mutation (insertion and deletion; indel) frequency of wild type and RGEN-transfected cells for each sgRNA

Target sites	Cells	Total counts	Mutation counts	Mutation ratio (%)
lpa2	Wild type	53898	21	0.00%
	gRNA1	52609	71	0.10%
	Wild type	42061	3	0.00%
	gRNA2	41760	172	0.40%
	Wild type	32156	2	0.00%
	gRNA3	34532	492	1.40%
	Wild type	334	0	0.00%
	gRNA4	316	0	0.00%

Table S4. Analysis of off-target effects in the wild type and *lpa2* mutant. Mutation frequencies at potential off-target sites of the *lpa2* gene-specific sgRNA2 were measured by targeted deep sequencing in the wild type and *lpa2* #1 and #2. Potential off-target sites that differed from the on-target sites by up to 4 nucleotides were selected. Different nucleotides between the on-target and off-target are highlighted in red.

**Target (5' to 3')**	**Cells**	**Total counts**	**Mutation counts**	**Mutation ratio (%)**
	Wild type	31640	0	0.00%
CAAGtGCTTTGGcTtcGAGACGG	*lpa2* #1	23022	0	0.00%
	lpa2 #2	14267	0	0.00%
	Wild type	145319	53	0.00%
gAAGGGCTgTGGgTCAGAGgAGG	*lpa2* #1	129094	63	0.00%
	lpa2 #2	22739	0	0.00%
	Wild type	274203	2190	0.80%
CgAGGGCTTTGGTgCcGgGATGG	*lpa2* #1	255326	1996	0.80%
	lpa2 #2	6752	16	0.24%
	Wild type	165055	747	0.00%
CgAGGGCTTTGGTgCcGgGATGG	*lpa2* #1	155692	622	0.00%
	lpa2 #2	8237	4	0.00%
	Wild type	45015	5	0.00%
CAAGGGCTTcGGTgCAGccAAGG	*lpa2* #1	41168	10	0.00%
	lpa2 #2	25300	2	0.00%
	Wild type	30798	0	0.00%
CAAGcGCTTTGcaTCAGAGgTGG	*lpa2* #1	29474	2	0.00%
	lpa2 #2	20140	2	0.00%
